# Dissecting Tumor Size Underestimation in Pancreatic Cancer: A Comparative Analysis of Preoperative Treatments

**DOI:** 10.1245/s10434-025-16917-6

**Published:** 2025-01-27

**Authors:** Kazuki Kobayashi, Yoji Kishi, Takazumi Tsunenari, Naoto Yonamine, Mikiya Takao, Takahiro Einama, Hironori Tsujimoto, Hideki Ueno

**Affiliations:** Present Address: Department of Surgery, National Defense Medical College, Tokorozawa, Saitama Japan

**Keywords:** Pancreatic ductal adenocarcinoma, Tumor size, Tumor size discrepancy, Preoperative image, Pathological specimens, Preoperative chemotherapy

## Abstract

**Background:**

Tumor size (TS) in pancreatic ductal adenocarcinoma (PDAC) is one of the most important prognostic factors. However, discrepancies between TS on preoperative images (TSi) and pathological specimens (TSp) have been reported. This study aims to evaluate the factors associated with the differences between TSi and TSp.

**Patients and Methods:**

We retrospectively analyzed patients with PDAC who underwent surgery at our institution between January 2010 and November 2023. TS discrepancy (TSD[%]) was defined as ([TSp − TSi]/TSp) × 100. Using logistic regression, we generated a receiver operating characteristic (ROC) curve to define the cutoff for TSi underestimation predicting clinical tumor (T) stage migration. Univariate and multivariate analyses were performed to evaluate predictors of TSi underestimation.

**Results:**

Of the 231 patients, 99 (42%) patients received preoperative chemotherapy. The ROC curve determined a TSD underestimation cutoff of 25.9%. The number of TSp > TSi cases was 185 (80%), and TSi underestimation was present in 117 (51%) patients. T stage migration rates were 76%, 26%, and 50% in clinical stage (c) T1, cT2, cT3, respectively, among the patients with chemotherapy, and 93%, 33%, and 14%, respectively, in those without chemotherapy. Multivariate analyses revealed that independent predictors of TSi underestimation were posterior surface invasion in the patients with preoperative chemotherapy and anterior surface invasion in those without chemotherapy.

**Conclusions:**

TS was more commonly underestimated than overestimated, and cT1 rarely corresponded to pathological (p)T1. The factors contributing to TSi underestimation differed between patients with and without preoperative chemotherapy. Therefore, these two groups should be considered separately for accurate TSi evaluation.

**Supplementary Information:**

The online version contains supplementary material available at 10.1245/s10434-025-16917-6.

Pancreatic ductal adenocarcinoma (PDAC) is the leading cause of death in developed countries.^1,2^ Among the various prognostic factors, including patient nutritional status^3^ and high preoperative carbohydrate antigen (CA)19-9 levels,^4^ ones of the most powerful are tumor size (TS)^5^ and resectability status. TS and resectability status are both applied to determine the T factor in the eighth edition of the Union for International Cancer Control (UICC) staging classification.^6^ T1–T3 are categorized solely on the basis of tumor size, and borderline resectable (BR) or unresectable (UR) tumors due to celiac axis, common hepatic artery, or superior mesenteric artery involvement are classified as T4. We have recently reported that TS is a prognostic indicator in resectable (R)-PDAC but not in BR-PDAC.^5^ Although selecting optimal treatment on the basis of TS is preferred, it has been reported that TS is frequently underestimated in preoperative computed tomography (CT) images compared with TS in pathology specimens.^7,8^ Preoperative therapy is increasingly becoming the preferred treatment strategy, and precise assessment of TS is expected to play a pivotal role in guiding treatment selection. However, the extent of TS discrepancy and the factors influencing the discrepancy remain unclear.

This study aimed to reveal the incidence and degree of TS discrepancies and to examine the factors associated with TS underestimation in preoperative imaging in patients who did and did not receive preoperative chemotherapy.

## Patients and Methods

### Patients

We retrospectively reviewed patients with PDAC who underwent radical surgery at the National Defense Medical College Hospital between January 2010 and November 2023. Patients with macroscopic noncurative (R2) resection or those who did not receive preoperative contrast-enhanced CT imaging were excluded because of the difficulty in accurate assessment of TS.

Indications for preoperative chemotherapy have changed over time. Preoperative chemotherapy was not administered routinely until 2014. Subsequently, only patients registered in the Prep-02/JSAP-05 trial received two cycles of gemcitabine with S-1 (GS).^9^ Since 2019, preoperative chemotherapy with GS has been implemented as a standard practice following the disclosure of the results of Prep-02/JSAP-05, showing significant survival benefits of NAC-GS treatment.^10^ L-OHP with CPT-11 with 5-FU/l-LV (FOLFILINOX) or gemcitabine with nab-paclitaxel (GnP) was used for BR/UR-PDAC. Patients were treated with gemcitabine or S-1 monotherapy at the physician’s discretion. Dose reduction or interruption during drug administration was determined by the attending physician.

### Study Design

**Fig. 1:**
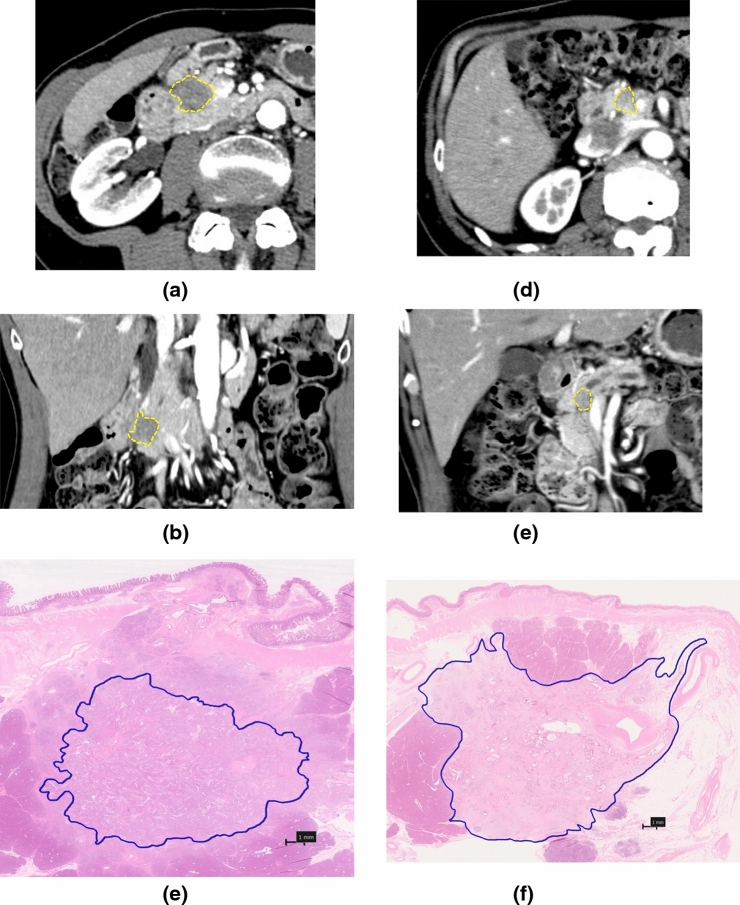
CT images of axial (**a**) and coronal (**b**) view of a 67-year old women who received preoperative chemotherapy. The maximum tumor diameter on CT is 15 mm. (**c**) Pathologic view of the maximal cut cut surface of the tumor of the same patient. The maximum tumor diameter is 14.9 mm, hematoxylin and eosin (HE stain), 35× magnification. CT images of axial (**d**) and coronal (**e**) image of a 73-year-old man who did not receive preoperative chemotherapy. The tumor is annotated with yellow dotted lines, maximum tumor diameter on CT is 15.8 mm. (**f**) The tumor maximally cut surface in pathology specimen. The tumor is annotated with blue line, HE stain, 35× magnification, maximum tumor diameter on pathologic specimen is 24 mm

The maximum TS was measured on preoperative CT images (TSi) and pathological specimens (TSp). In patients who received preoperative chemotherapy, TSi evaluated using CT after chemotherapy was compared with TSp. TSp was defined as the diameter of the contiguous tumor invasion area. The CT TS was retrospectively collected on the basis of diagnostic reports based on the evaluation by two radiologists. Additionally, all images were reevaluated by a single surgeon. The median TS determined from the radiology reports and the surgeon’s evaluation was defined as TSi. TSp was retrospectively derived from the pathology reports on the basis of assessments made by two pathologists. Although the pathologists were informed of the clinical course of the patients, the measurement of TS was performed independently from the radiologic assessment. TS difference (TSD) was calculated using the following formula: TSD (%) = ([TSp – TSi] / TSp) × 100. Figure 1 shows the annotated tumors on CT images and pathology specimens in patients with and without preoperative chemotherapy. For all patients, patient factors (age, sex, preoperative carcinoembryonic antigen [CEA] level, preoperative carbohydrate antigen 19-9 [CA19-9] level, resectability status [R/BR/UR], presence of preoperative chemotherapy, and pancreatic index [PI]) and histopathologic factors (location of the tumor [head or body/tail], histopathologic grade, anterior and posterior surface invasion, venous invasion, lymphatic vessel invasion, and TSp) were collected. Pancreatic index (PI) was calculated following a previous report as a diagnostic criterion for fatty pancreas (FP).^11^ Logistic regression was used to create receiver operating characteristic (ROC) curves to determine TSi underestimation and overestimation cutoff values that predict stage migration for cT<pT and cT>pT, respectively. The Youden index [=sensitivity-(1-specificity)] was used to identify the optimal cutoff value. The TSD corresponding to the highest Youden index was selected as the optimal threshold. TSD exceeding these cutoff values were defined as TSi underestimation (TSi_under_) or TSi overestimation (TSi_over_), whereas the cases with TSD within the two cutoff values were classified as TSi equivalent (TSi_equ_). In addition to the cases that the therapeutic effect of preoperative chemotherapy was complete response (CR; TSp = 0 mm), referring to the criteria for the revised Response Evaluation Criteria in Solid Tumors (RECIST) guideline (version 1.1)^12^, TSp-TSi < –5 mm was defined as TSi_over_. Univariate and multivariate analyses of the predictors of TSi_under_ were performed.

### Data Collection and Definitions

Clinical data, treatment, and pathological characteristics were collected. Serum CEA and CA19-9 levels were measured after chemotherapy in patients who received preoperative chemotherapy. Values above a quartile range (IQR) × 1.5 away from the first or third quartile were defined as outliers. In TSD, (first quartile − IQR × 1.5) was defined as TSi super-overestimation and (third quartile + IQR × 1.5) as TSi super-underestimation. Resectability status was classified according to the National Comprehensive Cancer Network (NCCN) guidelines.^13^ PI was calculated by dividing the CT value [Hounsfield Unit (HU)] in the pancreas by the CT value in the spleen.^11^ Histopathological and therapeutic effect of preoperative chemotherapy evaluations were performed in accordance with the Cancer Protocol Template for carcinoma of the pancreas by the College of American Pathologists (CAP).^14^ According to the CAP, histopathologic grade 3 was defined as poorly differentiated cancer, and therapeutic effect grade 3 was defined as poor or no response, with extensive residual cancer. Additionally, tumor invasion into the arterial system, such as the superior mesenteric artery (SMA) and common hepatic artery (CHA), and into the portal vein was evaluated histopathologically. Clinical and pathological stages were assessed according to the TNM classification system of the UICC, eighth edition.^6^

### Statistical Analysis

Continuous variables were expressed as median and interquartile range (IQR). The Mann–Whitney *U* test was used to compare continuous variables. Pearson’s chi-squared or Fisher’s exact test was used to compare categorical variables. Statistical analysis was performed using JMP^®^ software (SAS Institute, Cary, NC). Univariate and multivariate analyses were performed using logistic regression. All variables determined to have a significant trend (*p* < 0.10) in univariate analyses were candidates for multivariate analysis. The cutoff value for CA19-9 was set at 500 U/ml on the basis of the standard by International Association for Pancreatology (IAP).^15^ The cutoff value for PI was set at 0.70.^11^ Statistical significance was defined as *p* < 0.05.

## Results

### Patient Profiles

**Fig. 2:**
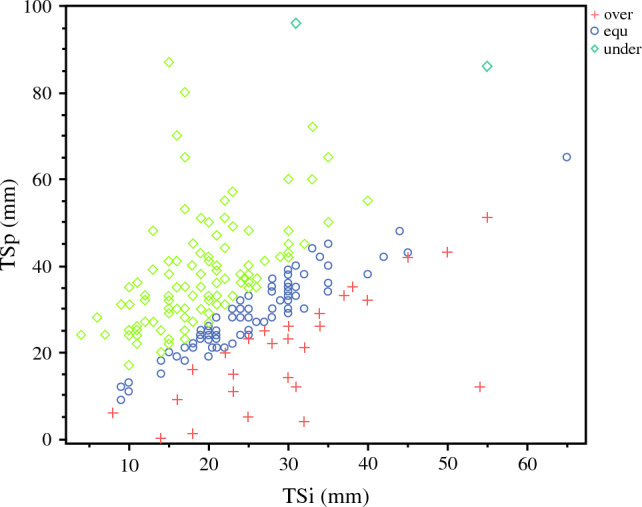
Scatter plot of TSi and TSp; TSi_over_, TSi_equ_, and TSi_under_ are indicated by a plus sign, circle, and square, respectively

**Fig. 3:**
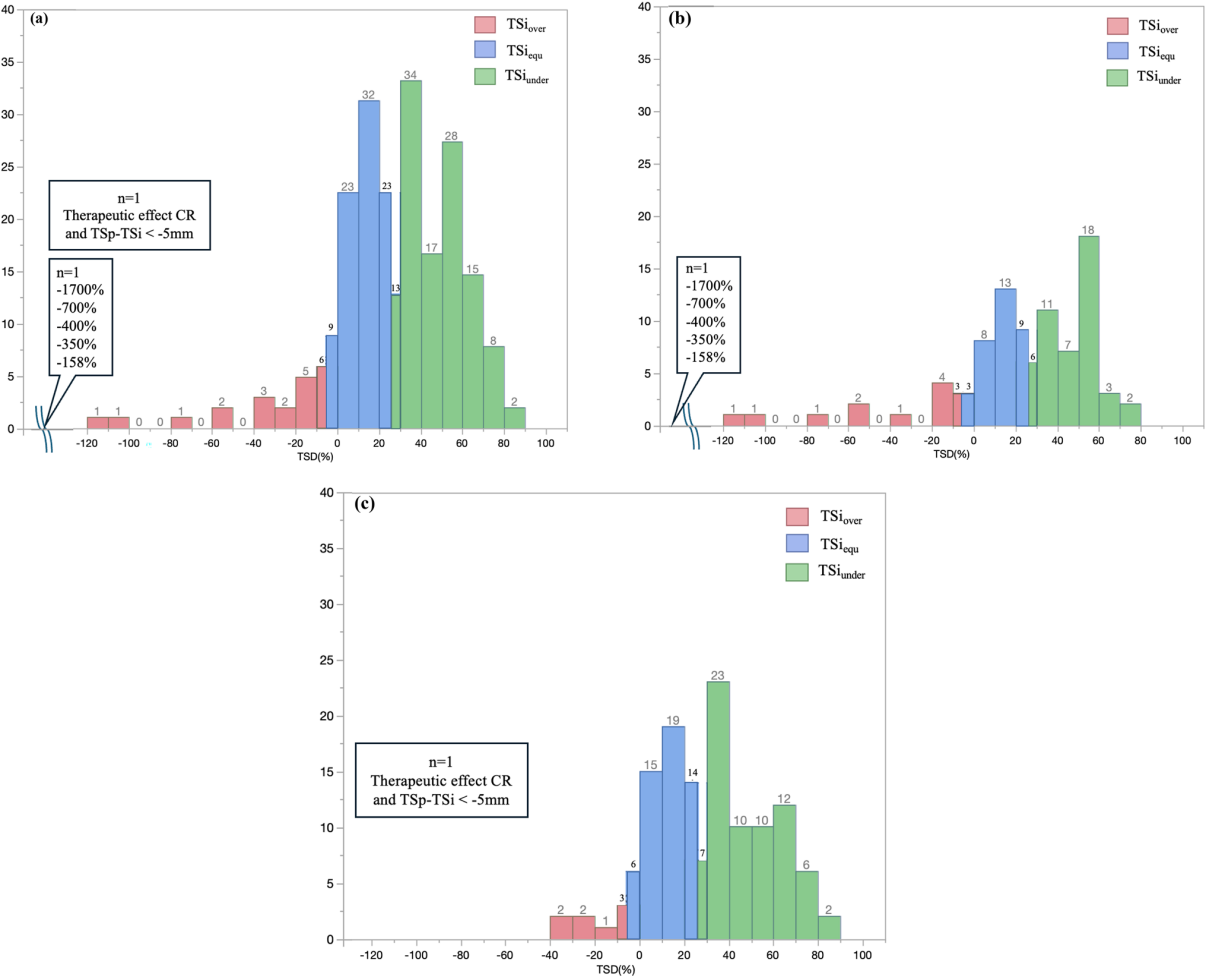
Histogram of TSD distribution in all cases (**a**), in those with preoperative chemotherapy (**b**), and in those without preoperative chemotherapy (**c**). TSi_over_, TSi_equ_, and TSi_under_ are shown in red, blue, and green, respectively. The number of patients with TSi_over_, TSi_equ_, and TSi_under_ were 15 (6%), 99 (43%), and 117 (51%), respectively, in the whole series, 11 (11%), 40 (41%), and 47 (48%), respectively, in those with chemotherapy, and 4 (3%), 59 (44%), and 70 (53%), respectively in those without chemotherapy.

A total of 241 patients underwent pancreatectomies during the study period; 2 patients who underwent R2 resection and 8 patients who could not undergo contrast-enhanced CT owing to renal dysfunction or history of allergy to the contrast agent were excluded from the study. The remaining 231 patients were included in this study. Patient profiles are summarized in Table 1. The median age was 71 years (IQR 25th–75th percentiles, 64–77 years), and 121 patients (52%) were men. The initial resectability statuses were R, BR, and UR in 173 (75%), 33 (14%), and 25 (11%) patients, respectively. A total of 99 (42%) patients received preoperative chemotherapy. The median PI was 0.82 (IQR 25th–75th percentiles, 0.72–0.94). R0 resection was achieved in 177 patients (86%). Using ROC curves, the TSD cutoff values for TSi_under_ and TSi_over_ were defined as 25.9% and −6.66%, respectively. The area under the curve (AUC) was 0.891 (95% confidence interval [CI] 0.851–0.932) for TSI_under_ and 0.783 (95% CI 0.646–0.922) for TSI_over_ (Supplementary Fig. 1a, b). Figure 2 shows a scatter plot of TSi and TSp. The median TSi and TSp values were 21 mm (IQR 25th–75th percentiles, 17–30 mm) and 31 mm (IQR 25th–75th percentiles, 24–39 mm), respectively. The median TSD was 25.7% (IQR 25th–75th percentiles, 9–46%). Figure 3a shows a histogram of TSD. The numbers of patients with TSi_over_, TSi_equ_, and TSi_under_ were 27 (12%), 87 (37%), and 117 (51%), respectively. Figure 3b, c show the TSD histograms for patients with and without preoperative chemotherapy, respectively. Among the patients who received preoperative chemotherapy, TSi_over_, TSi_equ_, and TSi_under_ were 18 (19%), 33 (33%), and 47 (48%), respectively. Among the patients who did not receive preoperative chemotherapy, TSi_over_, TSi_equ_, and TSi_under_ were 9 (6%), 54 (41%), and 70 (53%), respectively. The incidence of TSi_under_ was comparable between the patients with and without chemotherapy (*p* = 0.383). The number of patients with TSi super-overestimate (< − 46.5%) and TSi super-underestimate (> 101.5%) was ten and none, respectively (Supplementary Table 1). All ten patients with TSi super-overestimate received preoperative chemotherapy, with pathologic responses of grades 1, 2, and 3 in 2 (20%), 4 (40%), and 4 (40%) patients, respectively.

**Table 1:** Patent characteristics

	Study population *N* = 231
Sex, male/female	121 (52)/110 (48)
Age, years	71 (64, 77)
Preoperative CEA, U/ml	2.7 (1.9, 4.8)
Preoperative CA19-9, U/ml	81 (23, 304)
Resectability status R/BR/UR	173 (75)/33 (14) /25 (11)
Preoperative chemotherapy performed, yes	99 (42)
Chemotherapy regimen GS/GnP/FFX/Gem/S-1	71 (71)/13 (13)/11 (11)/3 (3)/1 (1)
PI	0.82 (0.72, 0.94)
TSi_over_/TSi_equ_/TSi_under_	27 (12)/87 (37)/117 (51)
Pancreatic head cancer	173 (75)
Histopathologic grade 1/2/3/other	51 (22)/125 (54)/43 (19)/12 (5)
Anterior surface invasion	111 (48)
Posterior surface invasion	197 (85)
Pathological T-factor 0/1/2/3/4	1 (1)/23 (9)/139 (60)/39 (16)/29 (4)
Pathological N-factor 0/1/2	50 (22)/92 (40)/89 (38)
Portal vein invasion+	89 (38)
Lymphovascular invasion+	208 (90)
Residual tumor *R*0/*R*1	197 (86)/34 (14)

Categorical variables are presented as *n* (%), whereas continuous variables are presented as medians (interquartile ranges)

*R* resectable, *BR* borderline resectable, *UR* unresectable, *Gem* gemcitabine, *GS* gemcitabine plus S-1, *GnP* gemcitabine plus nab-paclitaxel, *FFX* L-OHP plus CPT-11 plus 5-FU/l-LV, *PI* pancreatic index, *TSD* tumor size difference

### Frequency of UICC T Stage Migration

Table 2 presents the patients classified according to clinical T (cT) and pathological T (pT) categories among those with and without preoperative chemotherapy. In the patients who underwent preoperative chemotherapy, cT grade was lower compared with pT in 31 (76%), 10 (26%), and 1 (50%) of cT1, cT2, and cT3 cases, respectively. Among those who did not receive preoperative chemotherapy, the incidence was 51 (93%), 22 (33%), and 1 (14%) in cT1, cT2, and cT3 cases, respectively.

**Table 2:** Number of patients per clinical and pathological T stage by preoperative chemotherapy

Preoperative chemotherapy (−)					Preoperative chemotherapy (+)				
s	cT1 (*N* = 55)	cT2 (*N* = 68)	cT3 (*N* = 7)	cT4 (*N* = 2)		ycT1 (*N* = 41)	ycT2 (*N* = 38)	ycT3 (*N* = 2)	ycT4 (*N* = 18)
pT0 (*N* = 0)	0	0	0	0	pT0 (*N* = 1)	0	0	0	1
pT1 (*N* = 4)	4	0	0	0	pT1 (*N* = 19)	10	6	1	2
pT2 (*N* = 82)	35	46	0	1	pT2 (*N* = 57)	28	22	0	7
pT3 (*N* = 29)	11	11	6	1	pT3 (*N* = 10)	1	7	0	2
pT4 (*N* = 17)	5	11	1	0	pT4 (*N* = 12)	2	3	1	6
Stage matches (pT = cT)	4 (7%)	46 (67%)	6 (86%)	0 (0%)	Stage matches (pT = cT)	10 (24%)	22 (58%)	0 (0%)	6 (33%)
Stage underestimation (pT > cT)	51 (93%)	22 (33%)	1 (14%)	–	Stage underestimation (pT > cT)	31 (76%)	10 (26%)	1 (50%)	–

### Univariate and Multivariate Analyses for the Predictors of TSi Underestimation

Univariate and multivariate analyses of the factors associated with TS underestimation are presented in Tables 3 and 4, respectively. In cases with preoperative chemotherapy, multivariate analysis revealed that posterior surface invasion + (odds ratio [OR] 4.89; 95% CI 1.275–18.76; *p* = 0.020) was the independent predictor of TSi_under_. Histopathologic grade < 3 (OR 2.58; 95% CI 0.971–6.876; *p* = 0.052) tended to be associated with TSi_under_. Univariate analyses of the patients who did not receive preoperative chemotherapy revealed that anterior surface invasion (OR 2.80; 95% CI 1.373–5.709; *p* = 0.004) was a predictor of TSi_under_. Multivariate analysis was not performed because only one factor was extracted on the univariate analysis.

**Table 3:** Univariate and multivariate analyses of factors associated with TSi underestimation in patients who received preoperative chemotherapy

Factor	TSD > 25.9%	Univariate analysis		Multivariate analysis	
	*N* = 57 (47%)	*p*-Value	Odds ratio (95% CI)	*p*-Value	Odds ratio (95% CI)
*Sex*					
Female (*n* = 56)	28	0.566	1.26 (0.568–2.805)		
Male (*n* = 43)	19				
*Age, years*					
< 70 (*n* = 48)	26	0.197	1.68 (0.761–3.741)		
≥ 70 (*n* = 51)	21				
*Preoperative CEA, U/ml*					
≥ 2.7 (*n* = 60)	31	0.301	1.53 (0.680-3.469)		
< 2.7 (*n* = 39)	16				
*Preoperative CA19-9, U/ml*					
≤ 500 (*n* = 96)	46	0.623	1.84 (0.161–20.97)		
> 500 (*n* = 3)	1				
*Resectability status*					
R (*n* = 56)	29	0.327	1.49 (0.669–3.323)		
BR/UR (*n* = 43)	18				
*Therapeutic effect grade*					
3 (*n* = 74)	38	0.187	1.87 (0.736–4.781)		
0, 1, 2 (*n* = 25)	9				
*PI*					
≤ 0.7 (*n* = 27)	12	0.711	1.18 (0.486–2.875)		
> 0.7 (*n* = 72)	35				
*Tumor location*					
Body or Tail (*n* = 21)	10	0.988	1.00 (0.383–2.643)		
Head (*n* = 78)	37				
*Histopathologic grade*					
Others (*n* = 73)	39	0.050	2.58 (0.996–6.682)	0.052	2.58 (0.971–6.867)
3 (*n* = 26)	8				
*Anterior surface invasion*					
Yes (*n* = 51)	24	0.931	1.03 (0.470–2.278)		
No (*n* = 48)	23				
*Posterior surface invasion*					
Yes (*n* = 83)	44	0.019^†^	4.88 (1.296–18.43)	0.020^†^	4.89 (1.275–18.76)
No (*n* = 16)	3				
*Artery invasion*					
No (*n* = 87)	42	0.668	1.30 (0.384–4.435)		
Yes (*n* = 12)	5				
*Portal vein invasion*					
Yes (*n* = 44)	20	0.718	1.15 (0.522–2.561)		
No (*n* = 55)	27				
*Residual tumor*					
*R*1 (*n* = 11)	5	0.886	1.09 (0.311–3.856)		
*R*0 (*n* = 88)	42				

^†^Statistically significant

*CI* confidence interval, *R* resectable, *BR* borderline resectable, *UR* unresectable, *PI* pancreatic index, *TSD* tumor size difference

**Table 4:** Univariate and multivariate analyses of factors associated with TSi underestimation in patients treated without preoperative chemotherapy

Factor	TSD >25.9%	Univariate analysis	
	*N* = 70 (53%)	*p*-Value	Odds ratio (95% CI)
*Sex*			
Female (*n* = 54)	29	0.897	1.04 (0.522–2.098)
Male (*n* = 78)	41		
*Age, years*			
< 70 (*n* = 58)	33	0.431	1.31 (0.661–2.634)
≥ 70 (*n* = 74)	37		
*Preoperative CEA, U/ml*			
≥ 2.7 (*n* = 63)	36	0.366	1.37 (0.690-2.727)
< 2.7 (*n* = 69)	34		
*Preoperative CA19-9, U/ml*			
≤ 500 (*n* = 101)	56	0.317	1.51 (0.672–3.393)
> 500 (*n* = 31)	14		
*Resectability status*			
R (*n* = 117)	61	0.566	1.37 (0.460–4.115)
BR/UR (*n* = 15)	9		
*PI*			
≤ 0.7 (*n* = 21)	12	0.680	1.21 (0.475–3.122)
> 0.7 (*n* = 111)	58		
*Tumor location*			
Body or tail (*n* = 37)	21	0.597	1.23 (0.573–2.647)
Head (*n* = 95)	49		
*Histopathological grade*			
3 (*n* = 17)	12	0.128	2.35 (0.780–7.124)
Others (*n* = 115)	58		
*Anterior surface invasion*			
Yes (*n* = 60)	40	0.004^†^	2.80 (1.373–5.709)
No (*n* = 72)	30		
*Posterior surface invasion*			
Yes (*n* = 114)	60	0.817	1.12 (0.413–3.057)
No (*n* = 18)	10		
*Artery invasion*			
Yes (*n* = 17)	10	0.608	1.30 (0.466–3.678)
No (*n* = 115)	60		
*Portal vein invasion*			
No (*n* = 187)	47	0.750	1.12 (0.546–2.310)
Yes (*n* = 45)	23		
*Residual tumor*			
*R*1 (*n* = 23)	12	0.927	1.04 (0.423–2.565)
*R*0 (*n* = 109)	58		

^†^Statistically significant

*CI* confidence interval, *R* resectable, *BR* borderline resectable, *UR* unresectable, *PI* pancreatic index, *TSD* tumor size difference

## Discussion

This study observed an underestimation of TS in 51% of cases. Initially, the authors had hypothesized that the response to preoperative chemotherapy would make accurate estimation of tumor extent difficult. Unexpectedly, the proportion of underestimation was comparable between the patients with and without preoperative chemotherapy. However, chemotherapy should have caused morphologic change to some extent; thus, the authors analyzed the patients with and without preoperative chemotherapy differently in this study. The independent predictors of TS underestimation in patients who received preoperative chemotherapy were histopathologic grades other than grade 3 and posterior surface invasion; however, pathologic tumor response was not included. The predictor of TS underestimation in patients who did not receive preoperative chemotherapy was anterior surface invasion.

In this study, the criterion for TS underestimation was defined as TSD > 25.9%. As Fig. 1a shows, TSD was generally normally distributed, with the TSi_under_ population accounting for the largest proportion (51%). This trend remained similar in both subgroups of patients (with and without preoperative chemotherapy). Arvold et al. reported that TS was larger on pathology than CT in 84% of patients.^7^ In patients with cT stage underestimation, 42 received preoperative chemotherapy, while 74 did not. Bian et al. reported that accuracies of TSi for cT1, cT2, and cT3 disease were 61.02%, 79.41%, and 57.45%, respectively,^16^ although the results were not compared between patients who did or did not undergo preoperative chemotherapy. In this study, cT1 diagnostic discordance was very high at 93% in patients who received preoperative chemotherapy and 76% in patients who did not receive preoperative chemotherapy. The low diagnostic accuracy of the cT1 stage, regardless of preoperative chemotherapy, should be carefully considered during preoperative CT diagnosis. These results raise concerns about the need for improved diagnostic methods.

A noteworthy aspect of this study is that the extracted factors differed between patients who did and did not receive preoperative chemotherapy in the univariate and multivariate analysis. Factors that can directly influence the measurement of TS on CT images include anterior and posterior surface invasion. Components of the tumor microenvironment such as astrocytes, immune cells, and extracellular matrix are involved in pancreatic cancer progression.^17^ Peripancreatic fat is a major stromal component involved in extrapancreatic perineural invasion (EPNI), and its presence strongly influences prognosis.^17,18^ Concerning posterior surface invasion, since pancreatic head cancer frequently infiltrates the right posterior portion of the superior mesenteric artery (SMA),^19^ various approaches have been reported for dissection around the SMA for R0 resection.^20–23^ CT imaging evaluation of the sizeable pancreatic plexus 1 (PLX1) and extensive pancreatic plexus 2 (PLX2) originating from the celiac artery (CA) and SMA among the EPNI is well established.^24,25^ Khristenko, et al. reported that the diagnostic accuracy of PLX was 82.61%^26^. In accordance with previous literature, the results of this study suggest that posterior surface invasion was more accurately assessed in patients who did not receive preoperative chemotherapy than in those who received preoperative chemotherapy. The reasons for the inaccurate assessment of posterior surface invasion in patients who received preoperative chemotherapy could be as follows: (1) tumor progression due to waiting time before surgery and (2) partial response to preoperative chemotherapy obscuring the extent of extrapancreatic extension of the tumor.

It has been reported that TSi tends to be smaller than TSp in patients undergoing preoperative treatment for pancreatic cancer.^27,28^ Yang et al. reported that patients who underwent preoperative treatment tended to have larger TSp and exhibited larger TSD.^28^ A multivariate analysis of the preoperative chemotherapy group identified histopathological grades other than grade 3 as independent predictors of TSD > 25.9%. It has been suggested that tumors with low histologic differentiation are less likely to respond favorably to chemotherapy.^29^ Although pathologic therapeutic effect was not a predictor of TSi underestimation, we could make a hypothesis as follows. Tumors of histopathologic grade 1 and 2 are prone to respond to chemotherapy and result in promoted fibrosis and inflammation around the tumor cells.^30,31^ In pancreatic cancer, the tumor frequently induces fibrosis of the pancreatic parenchyma as a result of chronic pancreatitis due to the obstruction of the main pancreatic duct.^32^ Therefore, in these patients, tumor boundaries on CT images become obscure, and accurate TS assessment on CT imaging may be hampered. Noda et al. reported that preoperative chemoradiotherapy was a factor in TSi overestimation.^33^ In our study, patients with TSi super-overestimation were treated with preoperative chemotherapy. In addition, 60% of the patients had a therapeutic effect grade of 2 or less, indicating that TSi was overestimated in patients who responded well to preoperative chemotherapy. This finding suggests that there are cases in which areas of pathologically variable tumor cell disappearance are included in the TS on preoperative CT.

The definition of resectability status of pancreatic cancer has been based exclusively on anatomical factors, but was recently proposed to include biological, and conditional factors by IAP.^15^ As a biologic factor, CA19-9 value was suggested.^15^ In addition, TS has been increasingly recognized as a critical prognostic indicator, gaining attention as a significant biological parameter.^5,34^ In an era where preoperative treatment options are increasingly being chosen, TS assessed through imaging studies may serve as a crucial factor in determining the treatment regimen. The present findings highlighted the potential underestimation of TS by CT and may advocate for more intensive treatment, even in patients whose cancers are classified as cT1 pancreatic cancer. The authors of this study insist that the potential of underestimation of TS by > 25.9% should be considered when the initial CT images suggest anterior surface invasion, when the CT following preoperative chemotherapy indicates posterior surface invasion, or in patients with biopsy specimen showing histopathologic grade ≤ 2. However, the optimal TS cutoff for a biological factor of resectability status remains unclear and is expected to be addressed in further studies.

This study had several limitations. First, this was a single-center, retrospective study. Therefore, it consisted of a limited number of patients. Second, the TSp may have been underestimated, especially in cases of R1 resection. However, microscopic tumor exposure appeared to have a small effect on maximum TS. In addition, R1 resection may have been an effect of underestimation of the TS on preoperative CT imaging. This study aimed to examine the accuracy of preoperative CT in evaluating TS. Therefore, we did not exclude cases of R1 resection. Third, the TSp measured in the surgical specimens did not always correspond to the diameter measured on CT. This may have resulted in a low proportion of TSi_equ_. In preparation for an agreement on the measurement site, surgeons and histopathologists should collaborate and share the tumor size measurement method for each case in advance. Tumor size measurement methods should be the subject of future studies.

## Conclusions

The tumor size measured on preoperative CT images was smaller than that of the pathological specimen by more than 25.9% in 47% of patients with preoperative chemotherapy and 53% of patients without preoperative chemotherapy, and cT1 rarely corresponded to pT1. The factors contributing to the differences in tumor size differed between patients with and without preoperative chemotherapy; therefore, these two groups should be considered separately for accurate TSi evaluation on CT images.

## Supplementary Information

Below is the link to the electronic supplementary material.

Supplementary file1 (DOCX 17 KB)

Supplementary file2 (TIFF 27249 KB)

Supplementary file3 (TIFF 27249 KB)

## Data Availability

The datasets used and/or analyzed in the current study are available from the corresponding author upon reasonable request.
