# Supplementary material for: Dissecting Tumor Size Underestimation in Pancreatic Cancer: A Comparative Analysis of Preoperative Treatments
Source: Ann Surg Oncol. 2025 Jan 27;32(5):3593–602. doi: 10.1245/s10434-025-16917-6 (PMC11976789; doi:10.1245/s10434-025-16917-6)
Supplement: Supplementary file 1 — Supplementary file1 (DOCX 17 KB) [file 10434_2025_16917_MOESM1_ESM.docx]

Supplemental Table 1. Patient characteristics of TSi super-overestimation or super-underestimation

|  | TSi super overestimate  N=10 |
| --- | --- |
| Patient parameter | |
| Sex male/female | 4 (40) / 6 (60) |
| Age | 72 (63, 73) |
| Preoperative CEA, U/ml | 2.7 (1.7, 5.7) |
| Preoperative CA19-9, U/ml | 29 (8.9, 49) |
| Resectability status R/BR/UR | 4 (40) / 0 (0) /6 (60) |
| Preoperative chemotherapy performed, yes | 10 (100) |
| Therapeutic effect Grade 0/1/2/3 | 0 (0) / 2 (20) / 4 (40) / 4 (40) |
| PI | 0.83 (0.68, 0.94) |
| Histopathological parameter | |
| Pancreatic head cancer | 4 (67) |
| Histopathologic Grade 1/2/3/other | 0 (0) / 3 (50) / 3 (50) / 0 (0) |
| Anterior surface invasion, yes | 1 (10) |
| Posterior surface invasion, yes | 2 (20) |
| Pathological T-factor 0/1/2/3/4 | 0 (0) / 8 (80) / 0 (0) / 0 (0) / 2 (20) |
| Pathological N-factor 0/1/2 | 7 (70) / 2 (20) / 1 (10) |
| Portal vein invasion, yes | 1 (10) |
| Lymphovascular invasion, yes | 6 (60) |
| Residual tumor R0/R1 | 9 (90) / 1 (10) |
