# Supplementary figures and images for: Dissecting Tumor Size Underestimation in Pancreatic Cancer: A Comparative Analysis of Preoperative Treatments
Source: Ann Surg Oncol. 2025 Jan 27;32(5):3593–602. doi: 10.1245/s10434-025-16917-6 (PMC11976789; doi:10.1245/s10434-025-16917-6)

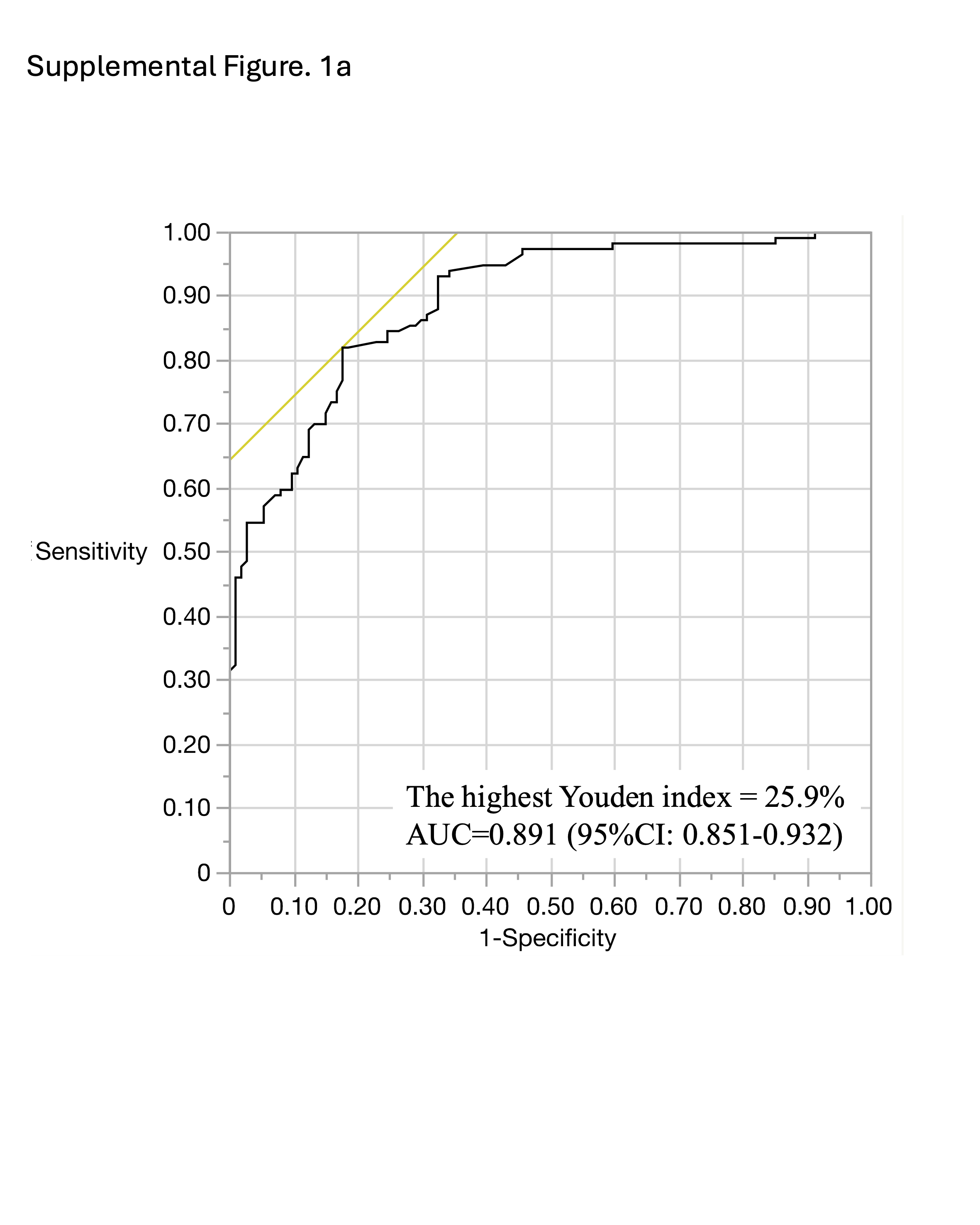

Supplement: Supplementary file 2 — Supplementary file2 (TIFF 27249 KB) [file 10434_2025_16917_MOESM2_ESM.tiff]

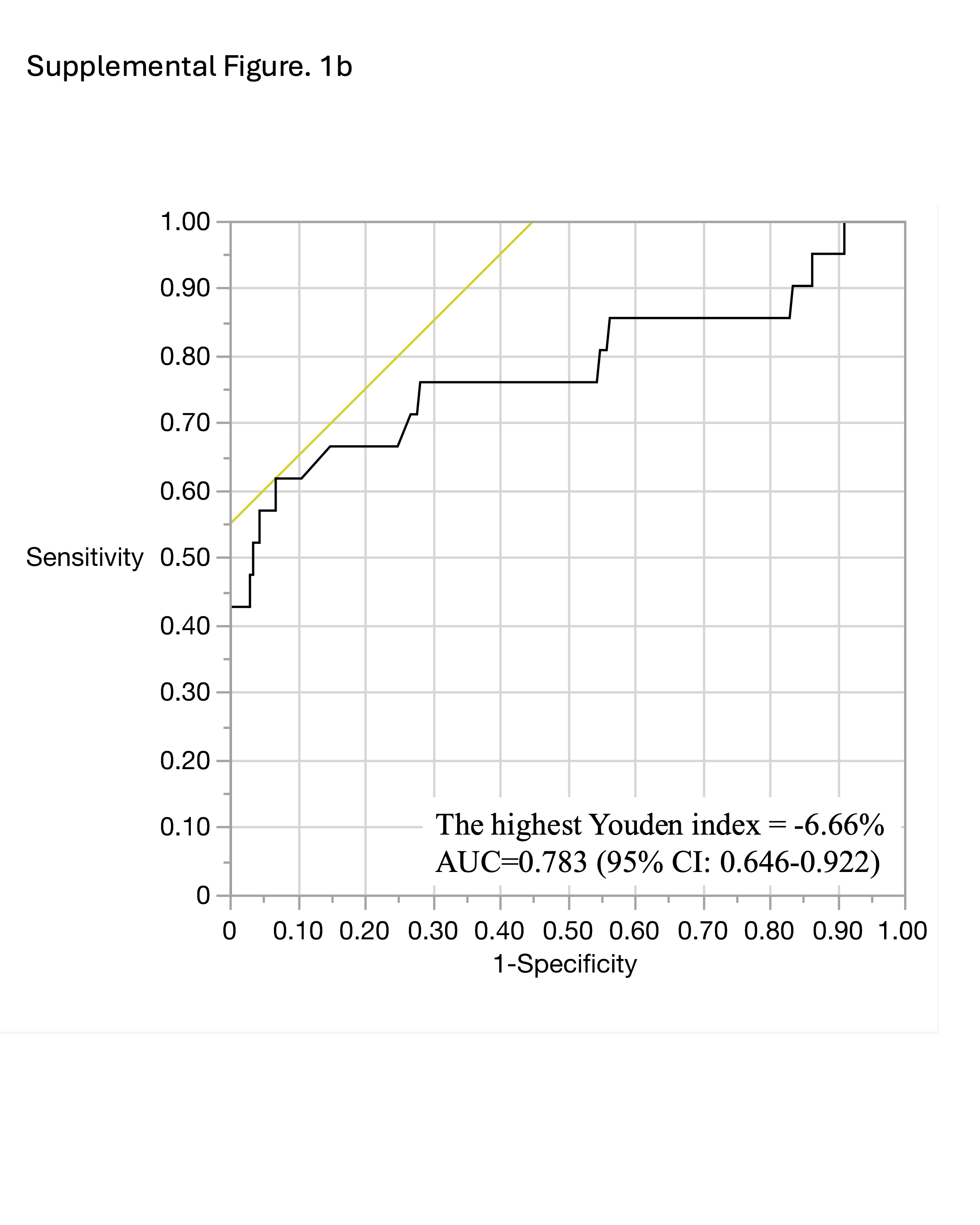

Supplement: Supplementary file 3 — Supplementary file3 (TIFF 27249 KB) [file 10434_2025_16917_MOESM3_ESM.tiff]
